# Psychedelic therapy without durable effects: A review and re-evaluation of controlled studies between 1965 and 1977

**DOI:** 10.1177/02698811261478606

**Published:** 2026-09-07

**Authors:** Torsten Passie, Michiel van Elk

**Affiliations:** 1 Hannover Medical School, Germany; 2 Dr. Senckenberg Institute for History and Ethics of Medicine, Frankfurt am Main, Germany; 3 Cognitive Psychology Unit, Institute of Psychology, Leiden University, The Netherlands

**Keywords:** psychedelic therapy, alcohol use disorder, efficacy, review, clinical trials, psycholytic therapy

## Abstract

Common wisdom in the psychedelic literature suggests that early controlled trials of psychedelic therapy were methodologically flawed. In this review we challenge this view and discuss 11 controlled studies that were conducted in the United States and Canada between 1965 and 1977 to evaluate psychedelic (peak) therapy for individuals suffering from alcohol and opioid use disorder and psychoneuroses. We provide a narrative review of the designs, methods, settings, treatment issues and results and show that these studies already adhered quite well to the regulatory and evidentiary standards of their time. These early psychedelic trials tried to effectively address common methodological problems that still threaten the validity of contemporary psychedelic clinical trials (e.g., regarding blinding and active control conditions). Our review indicates that despite a significant heterogeneity in the methods and research designs, most early controlled studies converge on the same conclusion: there are no durable improvements in the condition of patients treated with psychedelic therapy beyond a few weeks. We discuss implications for contemporary psychedelic science in terms of (a) patient selection (i.e., better prospects for non-chronic conditions), (b) the problems with a single-sided focus on mystical experiences as the primary mechanism of action, (c) the positive afterglow as a therapeutic window of opportunity and (d) the need for a strong psychotherapeutic framework to induce sustained therapeutic effects.

## Introduction

Since the 1950s psychedelic substances have been investigated as adjuncts to psychiatric therapies. Psychedelic therapy^1^ was developed and introduced by Canadian psychiatrists Humphry Osmond and Abram Hoffer in the mid-1950s, primarily for the treatment of chronic alcoholism (Oram, 2018). Treatment typically consisted of a few preparatory sessions, followed by one or two high-dose sessions with LSD or mescaline, sometimes followed by integration sessions. The therapeutic aim was the induction of a mystical “peak experience” (Maslow, 1961), which would make individuals with alcohol use disorder (AUD) aware of their “inauthentic self” and leave them with a profound sense of connectedness and better self-esteem to reduce symptoms and facilitate behavior change (e.g., Savage, 1968; Sherwood et al., 1968). Psychedelic therapy uses a suggestive and goal-directed preparation and setting. The flow and content of the experience are manipulated by the almost permanent use of eyeshades and headphones, specifically structured music and playlists, visual stimuli, artwork, religious symbols, etc., to direct the experience in a certain direction.

In the first decade of psychedelic therapy (1954–1964), before double blind and placebo-controlled research designs became the gold standard, no controlled studies were conducted, but promising clinical reports were published, mainly focused on the treatment of individuals with AUD (for narrative review, see: Piper et al., 2025). As was customary at the time, treatment studies were mainly based on pre-/post-measurements and numerous papers reported favorable results from such studies (e.g., Chwelos et al., 1959; Ditman et al., 1962; Fox, 1965; Jensen, 1962; MacLean et al., 1961; O’Reilly and Reich, 1962; Savage et al., 1964; Smith, 1964). However, despite initial enthusiasm among some researchers, considerable controversy existed in 1964 as to whether psychedelic treatment was useful and more effective than other contemporary treatments.

Since the mid-1960s, research with psychedelics in the United States became increasingly difficult and more controlled due to the 1962 Kefauver–Harris Amendments to the Federal Food, Drug, and Cosmetic Act, which introduced new regulatory requirements to prove both safety and therapeutic efficacy before a drug could be approved for clinical use (Hall, 2022). From that point on, LSD and related compounds could only be administered under an FDA Investigational New Drug protocol, and researchers needed to secure a license to possess and administer Schedule I substances, submit protocols, report adverse events and justify methodological choices to federal regulators (Bonson, 2018). These new regulatory and licensing requirements markedly increased the administrative burden and slowed new clinical studies, even before broader criminalization and the societal backlash curtailed psychedelic research at the end of the decade (Oram, 2018). Toward the end of the 1960s, concerns about increased recreational use of LSD put the research on these substances in a more critical perspective (Hartogsohn, 2020; Oram, 2018). However, because of the initially favorable reports of psychedelic therapy for chronic alcoholism (e.g., MacLean et al., 1961; Mogar and Savage, 1964) U.S. and Canadian research authorities, such as the U.S. National Institute of Mental Health (NIMH) and the Federal Drug Administration, remained willing to further research these promising treatments (see Bonson, 2018; Oram, 2018). Consequently, in the 1964–1977 period, at least 11 studies with improved methodologies were carried out by leading research groups to test the efficacy of psychedelic therapy for alcoholism, narcotic addiction and neurotic disorder – which are the focus of our narrative review.

We argue that there are important lessons to be learned from these studies for contemporary psychedelic science, which can be characterized by a revival of the psychedelic peak paradigm, in which a single high dose of a psychedelic is administered to induce therapeutic change. This current revival of psychedelic therapy is likely driven by different factors. In the late 1990s, a group of researchers gathered informally to discuss study designs to establish the positive effects of psychedelics on well-being (which resulted in the seminal paper by Griffiths et al., 2006). At the time, these studies had to be realized with minimal funding and were largely dependent on philanthropic donations. For instance, a review of the funding history of the National Institutes of Health between 2006 and 2020 indicated that in this period null psychedelic clinical trials received federal funding (Barnett et al., 2022). Likely causes for this are continued concerns about risks, federal laws preventing promotion of legalization of psychedelics and prioritization of grants for other types of studies. A survey conducted during the psychedelic revival indicated that even though among psychedelic researchers the funding success was perceived to be comparable to average NIH success rates, many perceived it was more difficult to get public funding for psychedelic therapy compared to other clinical interventions (Barnett, 2025).

This lack of funding likely favored cost-effective research designs, as the short-term model of psychedelic therapy facilitates efficient research designs, is more easily compatible with today’s regulatory standards and can be realized at lower costs. An initial research program, focusing on psilocybin-induced mystical experiences, was started in 2004 at Johns Hopkins University (Barrett et al., 2016; Griffiths et al., 2006). Since 2010 clinical research on psilocybin-assisted psychedelic therapy was initiated, focusing on the treatment of Major Depressive Disorder (MDD) and Treatment Resistant Depression (TRD) using PT. A first uncontrolled feasibility study showed a rapid improvement of symptoms (for a limited time span) in most TRD patients (Carhart-Harris et al., 2016). Other research using the psychedelic therapy paradigm showed promising results for the treatment of psychological distress in terminally ill cancer patients (Gasser et al., 2015; Grob et al., 2011). These findings have been replicated in two larger studies (Griffiths et al., 2016; Ross et al., 2016).

Together, these developments have resulted in a renewed interest in initiating clinical trials focusing on the efficacy of PT. These trends have also sparked a new wave of investments in psychedelic clinical trials by the psychedelic industry and new start-up companies focused on drug discovery and development, treatment and wellness centers and consumer goods (Aday et al., 2023). In turn, over the past decade, controlled psychedelic clinical trials have been conducted (e.g., Carhart-Harris et al., 2021; Davis et al., 2021; Goodwin et al., 2022), with mixed evidence for therapeutic efficacy in MDD and TRD patients, especially with respect to the durability of effects (van Elk and Fried, 2023). Other studies have focused on investigating the safety and efficacy of psychedelic therapy for the treatment of other clinical indications, such as AUD (cf., Bogenschutz et al., 2022) and obsessive-compulsive disorder (Moreno et al., 2006). Partly in response to the lack of strong and durable therapeutic effects (Meling et al., 2024) – but also due to challenges with the unpredictability of psychedelic experiences and difficulties with filing applications for intellectual property or for therapy – many psychedelic companies lost their stock value (Hager, 2025; Yoo and Sakopoulos, 2025). In terms of the Gartner-Hype cycle of new technological innovations, this has been described as the bursting of the psychedelic bubble and entering the “trough of disillusionment” (Yaden et al., 2022).

In this paper, we place this revival and decline of the psychedelic therapy paradigm in historical perspective. We take a step back by reviewing the early studies from the 1960s and 70s using psychedelic therapy for different clinical conditions: eight studies focused on individuals with AUD, two on narcotic addiction and one on chronic neurotic patients. Common wisdom in the psychedelic literature often seems to imply that the research of the 1960s and 70s was of questionable methodological quality (see for instance: Bonson, 2018, p. 598; Pollan, 2015). In this paper, we aim to correct this view by arguing that in response to the 1962 Kefauver-Harris Amendments to the FDCA (Oram, 2018), psychedelic researchers at the time started to think more carefully about how to improve their study design in order to meet the new regulatory standards. In fact, the early controlled psychedelic studies used designs that aimed to address many of the methodological challenges that we also face in contemporary psychedelic clinical trials (van Elk and Fried, 2023), for instance by using large sample sizes, different control conditions, different therapeutic interventions, active placebos and long-term (i.e., beyond 6 weeks) follow-up measurements (see Table 4 for evaluation and “Discussion” section for further discussion). Also, in contrast to the suggestion by Pollan (2015), we highlight that many controlled trials reported short-term improvements but no durable effects.

We thus believe it is valuable to provide an in-depth qualitative review of the design, therapeutic techniques and main findings of these early studies, which we present in “Review of the eleven controlled efficacy studies from 1965 to 1977” section. In the “Discussion” section, we reflect on the implications of the reviewed studies for contemporary psychedelic therapy and science.

## Review of the 11 controlled efficacy studies from 1965 to 1977

In this part, we outline the research design, populations treated, treatment conditions and techniques of 11 studies in some detail (the original studies can be found in the Supplemental Materials on the Open Science Framework; https://osf.io/ak3ze/overview).

### Selection of studies

The selection of studies was based on the following criteria: (a) having been published between 1965 and 1977, (b) including at least one other treatment condition as comparison and (c) the treatment and control group(s) were matched for potentially confounding factors (e.g., symptom severity, education, demographic variables). With “controlled studies” we mean a study that includes at least one other treatment condition to which the effects of the patients assigned to the psychedelic therapy were compared. Based on these criteria, we have included more studies than previous descriptive reviews, which were mostly limited to clinical trials focused on addiction (Abuzzahab and Anderson, 1971; Halpern, 1996; Mangini, 1998). Although there is some overlap, we have also included a different selection of studies than included in the meta-analyses by Krebs and Johansen (2012) and Sicignano et al. (2024) about the psychedelic therapy treatment of alcoholism (see Table 1 for comparison and Table 2 for overview of design). A recent narrative systematic review provides the most comprehensive summary of psychedelic-assisted therapy for substance use disorder (Piper et al., 2025) and is broader by including non-classical psychedelic substances such as ketamine, ibogaine and MDMA, as well as focusing not only on AUD but also on substance use disorder more broadly. We will reflect on the points of convergence and divergence between our review and previous meta-analyses in the “Discussion” section.

**Table 1.** Overview of controlled trials on psychedelic therapy for alcohol and opioid use disorder and neurotic disorder and whether the study was included in the meta-analysis by Krebs et al. (2012), Sicinago et al. (2024) and in our current narrative review.

Patient group	Year of publication	Included in Krebs et al. (2012)	Included in Sicinago et al. (2024)	Included in Passie & van Elk
Alcohol use disorder				
Smart Canada (*n* = 30)	1966	Yes	Yes	Yes
Cheek (*n* = 28 in LSD group)	1966	No	No	Yes
Hollister US (*n* = 72)	1969	Yes	Yes	Yes
Ludwig US (*n* = 176)	1969	Yes	Yes	Yes
Johnson (*n* = 95)	1969	No	No	Yes
Pahnke US (*n* = 117)	1970	Yes	Yes	No
Tomsovic US (*n* = 97)	1970	Yes	Yes	No
Bowen (*n* = 44)	1970	Yes	No	Yes
Kurland (*n* = 135)	1971	No	No	Yes
Rhead (*n* = 103)	1977	No	No	Yes
Bogenschutz (*n* = 95)	2022	No	Yes	No
Opioid use disorder				
Ludwig (*n* = 70)	1967	No	No	Yes
Savage (*n* = 74)	1973	No	No	Yes
Neurotic disorder				
Savage (*n* = 96)	1973	No	No	Yes

LSD: lysergic acid diethylamide.

**Table 2.** Overview of the studies included in our narrative review, including trial method, the psychedelic and dose administered and the control condition.

Patient group	Trial design	Psychedelic and dose	Control group and dose
Alcohol use disorder			
Smart Canada (*n* = 30)	R DB PC (for LSD)	LSD 800 mcg	Ephedrine sulfate 60 mg
Cheek (*n* = 28 in LSD group)	R SB PC	LSD 100 mcg initial dose LSD 200/300 mcg follow-up dose	No drug/placebo
Hollister US (*n* = 72)	R DB PC	LSD 600 mcg	Dextroamphetamine (80 mg)
Ludwig US (*n* = 176)	R DB (until LSD session)	LSD 3 mcg/kg	No drug/placebo
Johnson (*n* = 95)	R SB PC	LSD 300 mcg IV followed after 1 hour by a booster (depending on clinical insight)	(1) LSD without therapists(2) Methamphetamine(3) Treatment as usual
Pahnke US (*n* = 117)	R DB PC	LSD 450 mcg	LSD 50 mcg
Tomsovic US (*n* = 97)	R DB (until LSD session)	LSD 500 mcg	Treatment as usual
Bowen (*n* = 44)	R DB PC	LSD 500 mcg	(1) LSD 25 mcg(2) no LSD + therapy
Kurland (*n* = 135)	R DB PC	LSD 450 mcg	LSD 50 mcg
Rhead (*n* = 103)	R DB PC	DPT 75–165 mg	(1) treatment-as-usual(2) treatment-as-usual + intense psychotherapy
Bogenschutz (*n* = 95)	R DB PC	Psilocybin 25 mg/70 kg Session I Psilocybin 25–40 mg/70 kg Session II	Diphenhydramine 25 mg/70 kg Session I Diphenhydramine 50–100 mg Session II
Opioid use disorder			
Ludwig (*n* = 70)	R DB PC	LSD 2–3 mcg/kg	(1) Hypnodelic(2) hypnotherapy(3) psychotherapy
Savage (*n* = 74)	R DB PC	LSD 300–450 mcg	Treatment as usual
Neurotic disorder			
Savage (*n* = 96)	R DB PC	LSD 350 mcg	(1) LSD 50 mcg(2) Treatment as usual

R: random assignment; DB: double blind; SB: single blind; PC: placebo controlled; DPT: dipropyl-tryptamin; LSD: lysergic acid diethylamide.

The study by Cheek et al. (1966) was not included in previous meta-analyses for unclear reasons, even though it provided a controlled attempt to assess the effects of LSD in chronic individuals with AUD. The Johnson et al. (1969) study was not included in previous meta-analyses because it was single- instead of double-blind – we decided to discuss it here, as it provides an interesting example of how researchers improved their design to address common methodological problems beyond blinding. Bowen et al. (1970) and Rhead et al. (1977) were not included in previous meta-analyses because it proved impossible to retrieve the relevant statistical metrics from the reported results in the paper. Curiously, Kurland et al. (1971) was not included in previous meta-analyses, while it contained the finalized results from 135 patients, on which an interim report was published by Pahnke et al. (1970) with 117 patients, which *was* included in previous meta-analyses. For this reason, we do describe the Kurland and not the Pahnke study below. Ludwig et al. (1969) and Savage (1968, 1973) were not included in the previous meta-analyses as they focus on narcotic addiction and neurotic disorders – we decided to include them here as they were set up specifically to assess the efficacy of psychedelic therapy using a controlled design.

There are also studies that we did not include in our narrative review, but that were included in previous meta-analyses. Tomsovic (1970) was not included because it limited the inclusion of individuals with AUD to those with a diagnosis of schizophrenia, thereby presenting a complex case of comorbidity. We do acknowledge, however, the possibility that comorbidity might also apply to individuals with AUD more generally (Smith and Randall, 2012). We also do not review the recent Bogenschutz et al. (2022) study assessing the effects of psilocybin for the treatment of alcoholism, but we refer to this study in the “Discussion” section.

Thus, in this section, we first present studies on patients with alcohol dependence, then those on patients with narcotic dependence, and at last the one and only study on chronic neurotics. Studies are presented in chronological order, as they were often informed by the insights gained in previous studies. An overview of the study, its characteristics, including dosage and control conditions, is included in Table 3.

**Table 3.** Overview of reviewed controlled trials and to what extent they adhered to criteria for making more valid inferences about therapeutic safety and efficacy.

Patient group	Year of publication	Blinding	Blinding evaluated	Matched control groups	Control treatment identical	Active placebo	Blind rater	Follow-up (months)	Statistical conclusion validity	Risks, safety and adverse events	Conflict of interest	Selection bias	Mechanism of action evaluated	Other observations
Alcohol use disorder														
Smart Canada (*n* = 30)	1966	DB	Yes	Yes	Yes	Yes	No	6	High	Not reported	Low	Low	Pharmacological	
Cheek (*n* = 28 in LSD group)	1966	No	No	Yes	No; 6 weeks for control group, 4 weeks for LSD group	No	No	3, 6, 12	Medium	Not reported	Medium	Not reported	Attitudinal change	Embedded within AA program
Hollister US (*n* = 72)	1969	DB	No	Yes	Yes	Yes	No	2, 6	High	Not reported	Low	Not reported	Pharmacological	
Ludwig US (*n* = 176)	1969	DB	No	Yes	Yes	Yes	Yes	3, 6, 12	High	Low	Low/medium	Low	Psychotherapeutic/hypnodelic	
Johnson (*n* = 95)	1969	SB	No	Yes	Yes	Yes	Yes	12	Medium	Low	Medium/high	Medium	Mystical-type experience/transcendental state	
Bowen (*n* = 44)	1970	DB	No	No	Yes	Yes	No	12	High	Not reported	Low/medium	Low	Personality change	
Kurland (*n* = 135)	1971	DB	No	Yes	Yes	Yes	Yes	6, 12, 18	Medium/high	Not reported	Medium	Medium	Mystical-type experience + psychotherapy	
Rhead (*n* = 103)	1977	DB	No	Unknown	No	No	No	6, 12	High	Low	Medium	High	Mystical-type experience + psychotherapy	
Opioid use disorder														
Ludwig (*n* = 70)	1965/1967	DB	No	Yes	Yes	Unknown	No	0.5, 2	High	Low/medium	Low/medium	High	Psychotherapeutic/hypnodelic	
Savage (*n* = 74)	1966	DB	No	Yes	No	No	No	12			Medium/high			
Neurotic disorder														
Savage (*n* = 96)	1973	DB	No	Yes	No; 25% received a second LSD session; both LSD groups received more psychotherapy	Yes	No	6, 12, 18	High	Low/medium	Medium/high	Medium	Mystical-type experience/transcendental state	

A “High” indicates strong evidence for adherence, “Medium / high” / “Low / medium” moderate evidence and a “Low” flaws with respect to the evaluated criterion. “Not reported” indicates that the criterion could not be assessed based on the information reported in the paper.

DB: double blind; SB: single blind; LSD: lysergic acid diethylamide.

Throughout this review, we opted for retaining the historical diagnostic labels used in the original reports – in particular “narcotic addiction” and “(psycho)neurosis” – when describing how the original authors characterized their patient populations. The term “neurosis” was standard in the pre-DSM-III era (American Psychiatric Association, 1980) and is still used in the WHO’s ICD-10 (F40–F48; World Health Organization, 1992), but has largely been abandoned in contemporary scientific writing, missing a consensual definition. The term “narcotic” is pharmacologically and legally ambiguous. In pharmacology and medicine, “narcotic” is properly restricted to opioids because of their narcotic effects. Outside pharmacology, however, “narcotic” has carried a wider connotation. In the trials of “narcotic addiction” reviewed here, Savage et al. (1973) treated patients with heroin addiction; the participants of Ludwig and Levine (1965, 1967) are described as “narcotic drug addicts,” without further details. In line with contemporary nomenclature (American Psychiatric Association, 2013) we therefore refer to this population as individuals with opioid use disorder and use “narcotic” only when reporting the original terminology.

### Controlled studies with psychedelic therapy in patients with alcohol dependence

#### Cheek et al. (1966)

##### Participants and conditions

Twenty-eight inpatients with AUD were treated with two doses of LSD-25 (100 mcg and 200 or 300 mcg p.o.) and group therapy. Patients with psychosis and severe organic diseases were excluded. The control group received treatment as usual and was matched with respect to age, social status and education. The regular program lasted 6 weeks, the LSD program 4 weeks. Severity of alcoholism and attitude changes treatment were measured, with follow-ups at 3-, 6- and 12-months. Treatment rooms had nice furniture and prints on walls. Music was played for 30-min every hour.

##### Therapeutic approach

The treatment program was based on principles of the *Twelve Steps program of Alcoholics Anonymous (AA).* It included group and individual therapy, *AA* discussions, movies, pastoral counseling, occupational and recreational therapy. The goal of the LSD treatment was to reach a mystical experience leading to transformation of personality and behavior. The treatment was provided by a psychiatrist on call and a nurse, supported by a psychiatric technical assistant. Participants were instructed to provide a 15-min monologue (a few times per day) alone in a separate room, at times while looking in a mirror. In the afternoon, a 2.5-h group therapy session was organized with eight patients, including patients in the non-psychedelic group.

##### Measures

Follow-up interviews were conducted at 3, 6 and 12 months after treatment. The primary measure was the *Attitudinal Questionnaire (ATQ)*, which measures attitudes about illness and its consequences with a set of 32 items (e.g., “I blame myself for most of my problems” “My family would be better off without me”). Sobriety Status was measured by 3, 6 and 12 month follow-up interviews, using Rudie and McGaughran’s (1961) “essential reactive” scale for alcoholism, which is an interview form of a questionnaire containing 55 scorable items in eight areas like economic dependence, emotional dependency, application to reality tasks, relationship to friends and character traits.

##### Results

The average total change score on the ATQ was 81.0 points for the LSD group and 62.4 points for the control group at the end of treatment, a non-significant difference as established using a *t*-test. At 3 months, 60.7% of the LSD group and 44.1% of the control group stayed abstinent. At the 6- and 12-months follow-ups, the percentage of abstinent patients were almost the same in both groups, which was also true for improvement in family relations and work pattern.

#### Smart et al. (1966)

##### Participants and conditions

Studied were 30 “usual” inpatients with AUD, using a double-blind design. All patients had a long history of excessive drinking and previous unsuccessful treatments. Excluded were patients with psychosis and major cardiac or liver disease. Patients were randomly assigned to three groups, which were matched with respect to sex, education and marital status, occupation and drinking behavior. The LSD group was compared to one control group receiving ephedrine (a stimulant, as an active placebo) and a second control group exposed to an identical treatment, except for the drug sessions. Therapists who did the post-treatment therapy were “blind” about the condition the patients were treated with. Follow-up interviews were conducted with all patients at 6-months follow-up. All therapists had experience in LSD therapy and had at least one controlled LSD experience themselves.

##### Therapeutic approach

Patients were treated in a usual alcoholism treatment program. The drug treatment sessions were conducted in a one-bed plain hospital room, where patients were attached to their beds by a belt (“for security”). Without any information about the effects of LSD or ephedrine (!), the patients were injected intramuscularly with either 800 mcg LSD or 60 mg ephedrine. When drug effects wore off, patients were moved to a bed on a regular ward, to stay there for 1 week. Psycho- and occupational therapy facilities were available as well as psychiatric interviews and individual casework, plus a therapeutic community.

The study was already criticized at the time for (a) inconsistent presentation of results; (b) not using the standard of the psychedelic method (in a lot of respects); (c) inappropriate information of patients (no information about the drug’s effects); and (d) inappropriate setting (restrained on bed by belt). For further discussion, see Fadiman (1967); MacLean and Wilby (1967).

##### Measures

The following psychometric tests were used pre- and post-treatment: (a) The *Maudsley Personality Inventory (MPI)* and (b) an *Alcohol Use Questionnaire* (recording periods of abstinence, number of drinking days and amounts consumed).

##### Results

The results showed no differences between the three groups in any outcome measure. Evaluation of blinding showed that 19 of 20 drug sessions were guessed correctly, indicating effective unblinding.

#### Johnson et al. (1969)

##### Participants and conditions

This single-blind study tried to meet some criticism of the study by Smart et al. (1966) as put forward by MacLean and Wilby (1967) and Fadiman (1967). Therefore, two factors were altered: (a) the patient was guided by his therapist, after good rapport had been established; (b) Instead of a very high dose, a moderate dose was used.

Treated were 95 outpatients with AUD which were randomly assigned to four groups: (a) LSD (300 mcg i.v.) with a nurse, but no therapist present; (b) LSD (300 mcg i.v.) with therapist; (c) amobarbital-methamphetamine (3.5 g/30 mg, i.v. as an active control) with therapist; (d) treatment as usual without drug. Groups were matched for age, sex, marital status, education, type and length of employment and severity of drinking. Excluded were patients with psychosis and severe liver and cardiovascular or CNS disease.

##### Therapeutic approach

Treatments were carried out by social workers under psychiatric supervision. The treatment staff had themselves experienced LSD during training. The treatment room was simply furnished, quiet, shaded and restful. A restraint belt was used around the patient’s waist so that they could move around in bed but could not leave it. Six hours after drug administration, an injection of chlorpromazine was used to stop the LSD response and an oral sleep medication was given. After a night in the hospital, patients were interviewed by their therapist on the next morning.

##### Measures

The *Differential Personality Inventory* and an IQ test were used. A follow-up was done at 12 months by a blind rater unaware of the group to which the patient belonged. No standardized measures for judgment of pre-/post-betterments were mentioned. As was common practice at the time it can be assumed that “clinical interviews” were used. The authors wrote: “Responses were compared for pre- and post-differences in the areas of: (a) drinking-abstinence, drinking practices (frequency, length of benders, etc.), consequences of drinking (loss of jobs and friends, physical complications) and attitudes toward drinking (recognition and acceptance of need for sobriety); (b) employment; (c) accommodation; (d) social relationships; and (e) marital status.” An analysis of variance-repeated measures design was used to assess these pre- and post-measures.

##### Results

The study reports both pre-/post-differences and the differences between the treatment conditions. Analysis focused on abstinence and drinking pattern, consequences of drinking (loss of jobs and friends, physical complications), employment, accommodation, social relationships and marital status. In total, 87% of patients completed follow-up assessments. An analysis of variance-repeated measures design was used to assess pre-/post-measures. All treatment groups showed improvements on average, but no significant differences with respect to abstinence, alcohol-related problems and other parameters were detected between the groups.

#### Hollister et al. (1969)

##### Participants and conditions

Seventy-two patients with AUD were treated using a randomized, double-blind design to compare a single dose of LSD (600 mcg p.o.) to dextroamphetamine (60 mg p.o.) under otherwise identical treatment conditions. The two groups (36 patients per group) were matched for age, educational level and some social parameters. Excluded were patients with psychosis, severe affective disorders, CNS disorders and severe somatic diseases.

##### Therapeutic approach

No psychotherapy was provided. Patients received just minimal preparation and guidance for the session. The actual drugs were not named to the participants. A nurse present during the whole session was instructed to just answer questions and provide “necessary and brief reassurance.” The treatment rooms were “somewhat homier” than the usual hospital rooms, for example, equipped with a couch and casual chairs. Room were darkened during drug sessions and music was provided. On the day after drug session patients were sent home.

##### Measures

A new instrument was created for the study, the *Drinking Behavior Scale (DBS)*, to document drinking behavior, beyond the dichotomy “abstinent” or “non-abstinent.” Treatment outcomes were evaluated at three points: immediately after treatment, and at 2- and 6-month follow-ups. Evaluation interviews were done by a psychiatrist, who was blind to the treatment conditions. In addition, self-ratings using the *DBS* was collected.

##### Results

Around 75% of the patients completed the 2-months follow-up, at which the LSD group performed significantly better in terms of the *DBS*, while at the 6-month follow-up no significant differences between the groups could be found.

#### Ludwig et al. (1969)

##### Participants and conditions

The study used a double-blind design with different control groups. In total, 176 patients with AUD were randomly assigned to 4 groups receiving the following treatments: (a) *hypnodelic therapy* (hypnosis plus LSD and psychotherapy); (b) *psychedelic therapy* (LSD and psychotherapy); (c) *drug therapy* (LSD without therapist, instructed “to think about their problems”); (d) *no drug* (patients spent same time in treatment room “in contemplation and meditation,” instructed to take a “honest personal inventory” about their problems). During all LSD sessions, a staff member was continuously present. Following drug or no-drug treatment conditions, patients could work about problems in a group therapy program. Thirteen different psychiatrists worked as therapists in the study, to control the variable of differences in therapeutic skills. Therapists had no self-experience with LSD or hypnosis. Excluded were patients with overt psychotic and severe neurological or physical disease.

##### Therapeutic approach

The treatment for all groups was a 1-month intensive milieu therapy program for AUD, including five community meetings per week, small group therapy, lectures on alcoholism, *AA* meetings, occupational therapy and individual counseling. To minimize bias, patients were told in advance that all treatments were equally effective.

Patients in the LSD groups received 3 mcg/kg LSD p.o. in the morning. A staff member was present throughout the drug experience. According to the report of the authors, the “treatment lasted approximately three hours.” Thereafter, patients were brought to an “observation room” where they remained for the rest of the day (and overnight), in the presence of a specially trained nurse, offering “supportive therapy.”

##### Measures

The *California Psychological Inventory* and the *Psychiatric Evaluation Profile (PEP)* were used as the main assessment tools. Other measures were the *Drinking Follow-up Form* and the *Social Adjustment-Behavior Rating Scale (BRS)*, evaluating interpersonal relationships, employment and legal adjustment. Scores of the *BRS* domains were combined to create a “total adjustment score.” To minimize bias, the follow-up team was separated from the treatment team and was blind about the treatment conditions. Follow-ups were done as face-to-face interviews 3, 6, 9 and 12 months after discharge. The responder rate for the follow-up interviews was extraordinarily high (94.8%).

##### Results

Results showed a sharp increase in well-being during the first 3 months, followed by a plateau from 6 to 12 months. However, differences in well-being between the four groups were not significantly different. For an evaluation of the drinking pattern, a subscale of the *Adjustment-BRS* was used. Baseline to post-treatment *t*-tests revealed improvement for every group, while two-way analysis of covariance revealed no significant differences among the treatment groups. Analysis of covariance among the groups revealed no significant differences between outcome measures for any period, including the *PEP*.

#### Bowen et al. (1970)

##### Participants and conditions

This double-blind study by Bowen et al. (1970) included 44 patients with AUD. One group (*n* = 22) received LSD (500 mcg p.o.) and *Human Relations Training (HRT)*. The other group (*n* = 22) received 25 mcg LSD p.o. and *HRT*.

##### Therapeutic approach

Duration of hospitalization was 60 days for both groups. The *HRT program* (Blake and Mouton, 1955) presumes that alcoholics suffer mainly from an inability to cope because of low interpersonal skills. *HRT* provides exercises to teach interpersonal problem-solving skills. Patients were prepared for the LSD sessions in group lectures, which tried to establish positive expectations of the LSD experience. Patients selected the nurses sitting with them during the session. Nurses were trained to provide “positive support”. The treatment rooms were furnished with (non-hospital) tasteful furniture. Patients were told that they may receive either a large or a small dose of LSD dose but were not informed of actual condition assignment until a year later, after outcome data were collected.

##### Measures

Follow-up evaluations after 12 months were done in-person by a social worker, assessing abstinence, employment status, social organization affiliation, legal problems, incidences of delirium tremens or withdrawal and subsequent hospitalization. Patient’s adjustments were graded to be good (6–8 points), fair (3–5 points), or poor (0–2 points). To test for difference between the groups, Chi-square tests were used.

##### Results

Differences of all parameters used for evaluation were not significant between the two groups at the 12 months follow-up.

#### Kurland et al. (1971)

##### Participants and conditions

A double-blind trial was conducted with 135 patients with AUD. Patients were randomly assigned to (a) a high-dose LSD (450 mcg p.o.) group (*n* = 90) or (b) a low-dose LSD (50 mcg p.o.) control group (*n* = 45). The 7-week inpatient treatment was identical for both groups.

##### Therapeutic approach

Around 10 psychotherapy sessions were used to established rapport and to learn about the patient’s history, difficulties and psychodynamics. The goal of the LSD sessions was to achieve a “mystical psychedelic peak experience,” but there was also a significant amount of accompanying psychotherapy (ca. 20 h). The drug ses*s*ions were carried out in a room with a comfortable living room atmosphere, with sofa, easy chairs, rugs, drapes, pictures, flowers and hi-fi equipment. Nurse and therapists were permanently present during drug sessions. All staff members had self-experiences with LSD by training.

##### Measures

Psychometric instruments as the Minnesota Multiphasic Personality Inventory (MMPI), Personal Orientation Inventory (POI), PEP, Eysenck Personality Inventory (EPI)) were administered before and 1 week after LSD. A reduced battery of tests was given at the 6-, 12- and 18-month follow-ups. The social adjustment and alcohol consumption ratings at follow-ups, using the Global Adjustment Scale, were done by social workers blind to the treatment conditions.

##### Results

Despite matching and randomization, there were relevant demographic differences of the groups (e.g. twice as many divorces and more hospital admissions for alcohol problems in the low-dose group), suggesting more severe symptomatology and chronicity in the low-dose group. In addition, 52% of patients in the high-dose group had higher education, compared to 36% in the low-dose group.

A total of 89% of the patients participated in the 6-month follow-up, 80% in the 12-month, and 78% in the 18-month follow-up.

The psychometric instruments indicated improvement in both treatment groups, but no significant group difference was found immediately after treatment, and at the 12- and 18-month follow-ups.

#### Rhead et al. (1977)

##### Participants and conditions

In this randomized double-blind study, 103 male patients with AUD were enrolled. Excluded were patients with psychosis, CNS disease and severe cardiovascular or liver disease. Patients were randomly assigned to three groups: (a) Routine Hospital Treatment (RHT); (b) Conventional Therapy (CT) or (c) dipropyl-tryptamin (DPT)-Assisted Therapy. Patients in the RHT group received inpatient treatment as usual in a specific unit of the hospital (3 group therapy sessions per week, individual counseling, marital counseling, occupational rehabilitation and medical treatment). Patients in the CT group and in the DPT group received RHT, but the CT group received ca. 30 h, and the DPT group around 25 h of individual psychotherapy, plus the DPT sessions.

##### Therapeutic approach

Kurland’s team learned from a previous study of LSD treatment for AUD patients (Kurland et al., 1971) and developed the “extended psychedelic paradigm” (one high-dose session combined with a few low-dose sessions). The hypothesis was that multiple low-dose sessions (15–45 mg i.m.) might facilitate the resolution of psychodynamic material. This new technique was used in this study with the short-acting psilocybin-like substance DPT. Drug sessions were carried out in a comfortable living room, with carpet, sofa and easy chairs and music. A psychotherapist and a nurse were present throughout. Between the drug sessions, conventional psychotherapy sessions were scheduled. At first one or more low-dose sessions were applied, and at a later point, a high-dose DPT session (75–165 mg i.m.) was given.

##### Measures

The following tests were used: MMPI, POI, Psychedelic Experience Profile (PEP), the Outpatient Symptom Checklist, the Kempley Alcoholism Screening Behavior Inventory S Form 25 (KAS-25) and the Scale for Drinking Pattern. Assessments took place pre-treatment, at the end of treatment and at 6- and 12 months follow-ups.

##### Results

The authors write that all questionnaires used “contain 45 scales”. After study completion, statistical correlations of the 45 scales were calculated to identify group differences. Procedures for selection of the scales for correlations were not given. The authors postulate from their data sets that the DPT group performed better on some scales immediately after treatment. Not all outcome measures are reported in the manuscript, making it difficult to what extent there is selective reporting of results. At the at the 6- and 12-months follow-ups, in which 80–90% of their patients participated, no significant difference to the control groups was found.

### Narcotic addiction

#### Ludwig and Levine (1965, 1967)

##### Participants and conditions

A randomized double-blind study on patients with addiction to narcotic substances was conducted. Inclusion criteria were: age 20–35, not more than two previous hospitalizations, no serious physical illness—and a recommendation for psychotherapy by clinical staff. Seventy men with narcotic (i.e., opioid) addiction were randomly assigned to one of five groups: (a) *hypnodelic therapy* (LSD + hypnosis + psychotherapy); (b) LSD and psychotherapy; (c) LSD alone; (d) hypnotherapy alone; and (e) psychotherapy alone.

##### Therapeutic approach

The authors found previously that hypnosis can help to structure the LSD experience to increase its therapeutic benefits, termed “hypnodelic therapy” by these authors (Ludwig and Levine, 1967). In advance of the drug sessions, a 2–3-h preparatory sessions were held. In the groups treated with LSD, a dose of 2–3 mcg/kg p.o. was administered. Around 30 min before the onset of drug action, a hypnotic induction was applied in the hypnodelic therapy group to attain a hypnotic trance before the drug took effect. Toward the end of the session, the therapist attempted to summarize the important material discussed during treatment. Patients in the LSD plus psychotherapy group received some hours of psychotherapy in advance and after the LSD sessions, while the hypnotherapy group got some therapeutic hypnosis sessions and the psychotherapy alone got only some conventional psychotherapy sessions.

##### Measures

The *PEP* is a self-report inventory composed of four psychological domains (distress, distrust, optimism, competence). It was the main measure in this study, administered at baseline, 2 weeks and 2 months after end of treatment. It is remarkable that the study did not evaluate (report) drug use or relapse, but it is stated that all patients were abstinent during treatment.

##### Results

Pre/post measures showed improvements for all five treatment conditions. Sixty-nine of 70 patients were available for follow-ups. Patients receiving hypnodelic therapy had significantly greater improvement at the 2-week and 2-month follow-ups than any other group, in some scales of the *PEP*. No follow-up data for more than 2 months were reported, which is critical if it comes to a chronic disease as addiction to narcotics.

#### Savage et al. (1973)

##### Participants and conditions

This was a controlled study of psychedelic LSD psychotherapy with patients suffering from chronic heroin addiction, treated in an aftercare clinic which was called a “halfway house.” Patients were admitted to the outpatient clinic upon discharge from prison, with the treatment as part of their parole. Included were only parolees with 18 months of parole. Patients with organic brain damage, active liver disease, cardiovascular disease and psychosis were excluded.

Seventy-eight parolees were randomly assigned to two groups: (a) psychedelic therapy (one high-dose LSD session) during a 6-week residence in the half-way house or (b) receiving the outpatient clinic program with weekly group psychotherapy and daily urine monitoring. Patient characteristics indicated pretreatment between-group equivalence. The two groups were in identical treatment condition, but the LSD group had an additional stay in the clinic, which might have compromised the comparability between both conditions.

##### Therapeutic approach

During the patients’ 4–6 weeks residence at the halfway house, no formal therapeutic activities were scheduled. Psychedelic therapy consisted of 24 h of psychotherapy for a 5-week period, the climax of which was one high-dose LSD session (300–450 mcg p.o.) for the LSD group. After the psychedelic session, the patients received an additional week of therapy to “integrate the experience.” The control group did not stay on the clinic’s ward but participated in the regular outpatient clinic program, including daily urine surveillance and weekly group therapy.

##### Measures

The major outcome was abstinence as evaluated by daily urine tests and evaluative assessments for a total of 2 months following discharge. A *Psychedelic Experience Questionnaire* was administered after the LSD experience, which indicated that 80% of the patients achieved a “psychedelic peak experience.” Follow-up assessments of narcotic use and with the *Global Adjustment Rating Scale (GARS)* were carried out after 6 and 12 months.

##### Results

Thirty-seven participants in each group completed the entire treatment. Twenty-five percent of subjects in the LSD gr*o*up remained abstinent at the 12-months follow-up as opposed to 5% in the control group, what marks a significant difference. Social adjustment was evaluated with the *GARS* (10 is excellent, 1 is poor adjustment). No significant difference in the *GARS* scores was observed between the groups at the 12-months follow-up. However, the LSD group spent their first weeks in the halfway house and received double the amount of therapist’s hours as the control group, who only visited the clinic on an out-patient basis. This essential difference was clearly described in an earlier 1971 report of the study (Savage and McCabe, 1971), but appears obscured in the 1973 paper for unknown reasons.

### Neurotic disorders

#### Savage et al. (1973)

##### Participants and conditions

Effects of a single LSD session according to the psychedelic therapy paradigm were investigated in this randomized double-blind study with chronic neurotic patients. Ninety-six patients, diagnosed with “psychoneurosis” were treated, suffering mostly from symptoms of depression and anxiety (corresponding most closely to depressive and anxiety disorders in current terminology). Excluded were patients with organic brain damage and severe somatic disease. After screening, patients were randomly assigned to three groups: (a) a high-dose LSD group (350 mcg LSD p.o.); (b) a control group, receiving a low dose of 50 mcg LSD and (c) a control group receiving treatment as usual. Groups were matched for age and educational level. Authors report that random assignment failed to equally distribute patient variables to the groups.

##### Therapeutic approach

Patients completed a 3–5-week preparation phase with around 20 h of psychotherapy. Then an LSD session was conducted, with the patient’s therapist and a nurse present throughout. After the LSD session, patients remained in the clinic for at least 1 week, to receive further psychotherapy. After discharge, all three groups received additional psychotherapy sessions “for several months.” Some LSD patients of both groups treated with LSD were offered a second LSD session if it seemed “indicated” (25% received such additional treatment). The treatment as usual for the control group consisted of psychopharmacological medications and individual and group psychotherapy (90 min sessions, 3 per week). All therapists involved had self-experiences with LSD in their training.

##### Measures

The tests sued included *MMPI*, *EPI* and *PEP.* The *POI* was used to measure changes in self-actualization and values. Measures were administered at baseline and 6–8 weeks after end of treatment. Additional assessments, using the *Kast Adjustment Scale (KAS)*, occurred at 6, 12 and 18 months after treatment by a follow-up team, which was blind to the treatment conditions.

##### Results

A comparison shows that both LSD groups received around 20% more therapy hours and their follow-up treatment lasted longer on average (22 weeks in the high-dose LSD group, 19 weeks in the low-dose LSD group, 13 weeks in the conventionally treated group). Thus, the high-dose group received significantly more psychotherapy, which may have biased results. Pre/post measurement immediately after treatment (*MMPI*, *PEP*, *EPI*) showed a significant difference in favor of both LSD groups compared to treatment as usual. No significant difference was found between the two LSD groups. The follow-up assessments at 6, 12 and 18 months showed no significant difference between all three groups. This applies to the psychometric tests, but also to adjustment which was assessed by blind raters.

### History repeating: Leaving the psychedelic therapy paradigm behind

How did the researchers involved in the early clinical reviewed above, respond to the rather disappointing results? The “Discussion” section of Ludwig et al. (1969, p. 66) reads as follows:

It would be professionally gratifying to report significant results for the hypnodelic treatment technique, as well as for the other LSD treatment conditions, not only for the purpose of cross-validating our prior findings with drug addicts but also for making some contribution to the treatment of alcoholism. Unfortunately, our conscious wishes must yield to the overwhelming evidence that none of the LSD treatment procedures produces any treat therapeutic benefit than can be realized by the “no therapy” condition in the context of the general ward milieu program.

Most historical accounts of early psychedelic therapy research (see for instance: Pollan, 2019) attribute the decline of psychedelic research in the late 1960s mostly to the legal ban on the use of these substances, because the use of psychedelics by laymen became a mass phenomenon. While these factors likely contributed to the decline of research on PT, the lack of favorable results of the (methodologically refined) later studies has likely played a significant role (see also: Hall, 2022).

We shortly review some historical developments relevant to this issue. The leading research in the field of psychedelic therapy since the early 1960s was a group at the *Maryland Psychiatric Research Center (MPRC)* in Baltimore (cf., Yensen, 1994). The *MPRC* group included major proponents of PT, such as Albert Kurland, Walter Pahnke, Charles Savage, Sanford Unger, Stanislav Grof and William A. Richards. The group’s initial optimism about the efficacy of psychedelic therapy was tempered through the methodologically refined studies, including their own, which failed to produce significant results in favor of PT. An exception was the study of Savage and McCabe with individuals with narcotic use disorder (Savage et al., 1973), which produced significant results in favor of PT. But different amounts of therapy were offered to the control and the psychedelic group, and other significant differences in setting variables question the validity of their results.

The group at the *MPRC* responded to the negative findings by developing a new therapeutic approach, discussed by a research psychologist at the *MPRC*:

although improvements can be observed when a strong mystical-religious peak experience has occurred, in the study of individual cases . . . it became clear that many of the “cured” alcoholics . . . were not absolutely “cured”. . . . [This] suggests that Psychedelic Peak Therapy could be improved by incorporating an analytic approach. Recent work at the MPRC utilized what might be termed a psychedelytic or extended psychedelic paradigm. This includes . . . an increased number of [sometimes lower-dosed] drug sessions and an equal emphasis on the psychodynamic levels of consciousness and the ego transcendentals of the mystical experience. . . . [to] combine the positive aspects of psychedelic and psycholytic approaches (Yensen, 1975; 51/52).

Indeed, the last studies at the *MPRC* employed a series of drug sessions and much more psychotherapy. For instance, an open-label study by Yensen et al. (1976) with neurotic outpatients used three to four sessions with the entactogenic drug MDA (methylenedioxy-amphetamine), embedded in a 6-month period of conventional psychotherapy (around 50 sessions). Another open-label trial used eight low-dose sessions with the short-acting psilocybin-like compound DPT and 60 h of psychotherapy (Soskin et al., 1973). After these exploratory studies yielded good results, a controlled trial with the “extended psychedelic paradigm” using DPT was conducted, but did not show significantly better results than the control group (Rhead et al., 1977).

A subsequent grant application of the *MPRC* group for a next research study states:

Our collaborators agree that we have left the classical psychedelic orientation . . . behind us. . . . [It] is clear that the peak experience is not the “sine qua non” for successful behavior change. Although patients who experience a peak experience . . . place great value on the memory, it does not appear to be a guarantee of relief from disruptive symptoms or of lasting personality maturation. Therefore, the somewhat magical one-session model that we have mainly worked with over the last ten years is now seen as inadequate. There is a consensus that in our endeavor to find a brief form of depth psychotherapy we have tended to set unrealistic limits on the duration of treatment. (Kurland et al., 1975, p. 3)

The grant application involved a study proposal (that was never conducted) intending to compare *Psychedelytic* Therapy with classical *Psycholytic* Therapy (Buckman, 1967; Passie et al., 2022; see also Table 4) to gain insight into therapeutic mechanisms. Such a study has to-date not been conducted but appears critical and timely.

## Discussion

We reviewed controlled studies about the efficacy of psychedelic therapy in the 1965 and 1977 period, the first phase of broader controlled research on the therapeutic use of psychedelics (Passie, 1997; Yensen, 1994). These studies built on earlier uncontrolled studies reporting favorable results in the early 1960s and undertaken to gather more robust scientific evidence for the efficacy of psychedelic therapy using more refined methodologies. Below (a) we argue for a re-evaluation of the early controlled studies in light of current methodological recommendations for psychedelic clinical trials, (b) we discuss the implications for the efficacy of PT, (c) place our findings in the context of meta-analytic and historical reviews and (d) we discuss implications for contemporary psychedelic therapy.

### A re-evaluation of the early controlled studies of psychedelic therapy

Observations made by researchers of the reviewed studies reflect that they were well aware of methodological problems that limited conclusions about the therapeutic effects of psychedelic therapy already at that time. For instance, the discussion section of Hollister et al. (1969) reads:

Almost all studies can be criticized on the usual grounds: absence of controls or random assignment to comparison treatments, failure to use blind techniques, failure to account for nonspecific factors in treatment programs and inadequate follow-up procedures.

These methodological issues show significant overlap with the methodological problems that we still witness in contemporary trials using the psychedelic therapy paradigm (Aday et al., 2022; Muthukumaraswamy et al., 2021; van Elk and Fried, 2023). In Table 4 we provide an overview of the different criteria that we recently listed to evaluate the quality and rigor of contemporary psychedelic clinical trials (van Elk and Fried, 2023). Based on the limited available evidence found in the papers, we have indicated to what extent the reviewed clinical studies from the 1960s and 70s met or failed those criteria.

**Table 4.** Comparison of the psychedelic therapy and the psycholytic therapy paradigm with respect to dose, intended effects, thereaputic approach and patient groups for the use of hallucinogens in psychiatry.

Therapeutic approach	Psychedelic therapy	Psycholytic therapy
Dosage	High doses of LSD (400–1500 µg)	Low doses of LSD (30–200 µg) or psilocybin (3–15 mg)
Intended effects	Cosmic-mystical experiences	Dreamlike symbolic imaginations
	Self-transcendence	Regressions
	Ecstatic feelings of happiness	Transference phenomena
Theoretical foundation and purpose	Without foundation in classical psychological theories *(Transpersonal Psychology)*	Psychoanalytic theory
		Activation and deepening of the psychoanalytic process
Number of sessions	A single overwhelming experience	Numerous sessions required
Therapeutic approach	Highly suggestive preparation	Analytical discussion of the material experienced in individual and group sessions
	Use of specific environmental conditions	
	Structuring music	
	No detailed discussion of experiences	
Influence on experience	Strong	Limited
	Active accompaniment by sitter	Simple, comfortable setting
	Eye mask and headphones	No eye mask/headphones
	Strongly structuring music	Reserved sitter
	Comfortable setting with visual stimuli	Quiet background music
		No interpretations
	Supportive interventions and interpretations	Hardly any physical interactions
	Supportive physical interventions	
	Consolidation of the peak experience	
Therapeutic processing	Reality adaptation not primary	Reality check and attempt to adapt experiences to everyday life
Therapeutic goal	Symptomatic healing through behavioral change	Healing through personality restructuring in the sense of a maturation process
Patient groups	Alcohol use disorder	Classic indications of psychotherapy:
	Patients with life-threatening illnesses	
	Substance abuse disorder?	Neuroses
		Psychosomatic illnesses
		Personality disorders
	Psychoneuroses?	Sexual disorders

LSD: lysergic acid diethylamide.

Our review indicates that the reviewed early studies used blinding, control conditions (e.g., treatment as usual, high vs low doses, active placebos) and matched control groups (e.g. in terms of demographic background and symptom severity). The studies also tried to manipulate the therapy component (e.g., by using more than one control group; comparing different therapeutic approaches in different control groups). All studies included long-term follow-up measurements, usually up to 6 or 12 months and beyond – a timeline that is hardly ever met in contemporary psychedelic therapy trials. Establishing the efficacy of psychedelic therapy beyond 6 weeks after the intervention is especially important given the cyclic nature of most chronic conditions (for discussion see: Whiteford et al., 2013). Still, we note that while a 3-month improvement in problematic drinking is typically considered evidence for initial remission (Hagman et al., 2022), even such a short-term improvement can be clinically relevant and meaningful – especially in light of the continuing care model of substance use treatment (Proctor and Herschman, 2014). From a statistical point of view the sample sizes were reasonable and comparable and sometimes larger than observed for contemporary psychedelic therapy trials. Compared to today’s standards, statistical reporting was minimal (most studies reported descriptives and some formal statistical tests). However, given that most studies reported null-results with respect to durability, we infer that the statistical conclusions are in line with the data. A notable exception is the study by Rhead et al. (1977) which reports an extensive number of post hoc analyses on sub-scale scores and sub-group analyses (e.g., on p. 254 it reads “none of the 54 analyses of variance was significant” – without properly correcting for multiple comparisons). Hence this study does not provide a consistent pattern of results – except that at face value the groups did not differ significantly on key clinical outcome measures at 6- and 12-months post-treatment.

As was customary at the time, hardly any study explicitly reports on potential untoward responses to LSD, that is, on (serious) adverse effects. However, Ludwig and Levine (1965) report on physical side-effects such as nausea, headache and dizziness that were common; psychological side-effects that were mentioned included transient anxiety and insomnia. The paper notes that three patients showed adverse reactions in the weeks after the psychedelic therapy trial, including increases in psychopathology (requiring hospitalization), psychotic ideation as well as increased anxiety and depression. Despite this, the paper concludes: “In general, it was felt that the number of undesirable effects was minimal and, for the most part, could be managed with routine hospital procedures.” Savage et al. (1973) also report on one patient who developed a psychotic episode and recovered after psychotherapy and antipsychotic medication. The author puts this in perspective by stating that of the “nearly 500 diverse patients treated by the authors since 1964, only one other similar reaction occurred . . ..”.

It is difficult to evaluate to what extent there was a bias in the selection of patients. In contemporary psychedelic therapy trials, selection bias happens when participants signing up for psychedelic therapy trials have exceedingly high expectations about psychedelics as a “miracle cure,” often exacerbated by the popular press and/or positive prior experiences with psychedelics (Aday et al., 2022). Some of the reviewed papers do not provide sufficient information to make inferences about the problem of selection bias of patients. For the studies conducted at research institutions that were known at their time for running clinical trials with different substances, including psychedelics (e.g., the *MPRC*), we can infer that patients who self-enrolled for treatment knew they might participate in PT. Johnson (1969) reported a strong selection prior to enrollment (74 dropouts) and after intake (66 dropouts), for unknown reasons. Similarly, Ludwig and Levine (1965) reported that 35% of patients were rejected prior to the study “for having failed one or both suggestibility tests,” 7% did not want to participate and 6% were not accepted for “miscellaneous reasons” – suggesting a bias toward selecting patients who might favorably respond to PT. For other studies (e.g., Rhead et al., 1977) selection bias happened after enrollment, due to higher attrition in the non-psychedelic compared to the psychedelic arms (likely due to disappointment/nocebo-effects for control patients), which might have biased the results. Other studies tried to minimize selection bias through selective disclosure, for example, by informing patients that all treatment arms were deemed equally effective (Ludwig et al., 1969) or by not informing them that they might get a psychedelic drug (Hollister et al., 1969).^2^ A survey among 19 psychedelic researchers in 1966 – shortly after a sensationalistic publication in Life Magazine about the alleged risks and adversities triggered by LSD – indicated that indeed alarmist media-reporting negatively affected the willingness of volunteers to participate in psychedelic trials (Dahlberg et al., 1968). It also increased the anxiety of both the patients and the ambivalence and cautiousness of the personnel conducting the research, which in turn might also have had a negative effect on selection bias and the outcomes of the studies conducted at that time. On the other hand, Yensen and Dryer (1992) have argued that the degree of enthusiasm of the investigator could actually favorably influence the therapeutic outcome from LSD treatment in the early trials.

Finally, we classified the primary hypothesized mechanism of action based on the information provided in the paper. We made a distinction between studies that primarily relied on a biochemical approach (i.e., based on the assumption that the pharmacological effects of psychedelics would suffice to induce therapeutic change), studies that focused on the psychotherapeutic mechanisms underlying psychedelic therapy or studies aimed at inducing a “psychedelic peak experience.” We note that in contemporary psychedelic science, we witness a similar debate about the relevance of subjective experiences compared to the biochemical effects of psychedelics for therapeutic efficacy (Olson, 2020; Yaden and Griffiths, 2020) – a debate that as-of-yet appears to be unresolved.

According to today’s standard, there are also methodological issues which have not been addressed at all, such as reporting on the integrity of blinding (although one study reported blinding efficacy), measurement of the patients (and therapist’s) expectancy effects, measurement of nocebo reactions in the control groups (i.e., disappointment of subjects in placebo groups may accentuate group differences), evaluation of possible biases of therapists and potential bias produced by (self-)selection of patients. In addition, these studies face the same problem as many clinical trials, namely that the inclusion criteria tend to exclude more severely ill patients and/or those with certain comorbidities. Such exclusion criteria lead to the treatment of “unreal populations” as the severely ill patients will be excluded from the trial. Others have noted similar risks of bias in their review of the early controlled trials (Piper et al., 2025). Although we did not formally assess publication bias (i.e., positive findings typically have a higher likelihood of being published than null findings), we do note that the studies we reviewed stand out mostly in terms of their reporting of lack of significant long-term effects. A recent meta-analysis of the effects of psilocybin for depression (Metaxa and Clarke, 2024) found no evidence for publication bias for remission rates, but an asymmetry in the funnel-plot was observed for response estimates. However, this effect might be related to the possibility that most smaller studies with lower sample sizes (and hence more imprecise effect size estimates) are conducted at the early phases of new psychotherapeutic interventions, in which more motivated participants may have been included or the therapy may have been provided more effectively (for discussion, see also: Meling et al., 2024).

Based on our narrative review, we argue the methodological quality of the reviewed studies was more sophisticated than their reputation in the literature (Bonson, 2018; Pollan, 2015). No single study implemented all quality criteria that we currently deem necessary for conclusions about safety and efficacy. But considering the standards of their time, most reviewed studies incorporated some methodological refinements in their study design (see Table 4).

### Implications for the durability of effects of psychedelic therapy

We argue that the rather uniform result of these heterogeneous studies (i.e., no significant improvement left after a few weeks up to more than 3 months) point in the same direction: that is, while some of the studies of psychedelic therapy reviewed here have demonstrated positive evidence for a short-term improvement in patients with AUD, addiction to narcotics and psychoneuroses, the treatment effects were often not durable and disappeared after a few weeks or months.

It appears that the lack of durability is not caused by the specific psychedelic therapy protocol used, as the studies employed different therapeutic approaches (and preparations) before and during the psychedelic therapy sessions. Given that the long-term benefits of psychedelic therapy failed to hold up against proper control groups, it seems that at the time the psychiatric community already largely dismissed psychedelic therapy as being ineffective for chronic mental health conditions (Oram, 2018). The time-limited character of improvements raises more general skepticism about the claim that a single drug experience combined with minimal therapeutic preparation can lead to a lasting improvement or personality transformation. This claim has been at the heart of the psychedelic peak therapy paradigm, whereby a single psychedelic-induced mystical-type experience is hypothesized to underlie the therapeutic effects of PT. This paradigm dominates contemporary psychedelic therapy trials methodology, which – partly inspired by concerns about cost-effectiveness – also typically use a single or a few high-dose psychedelic sessions with minimal preparation and integration (for critical discussion, see e.g., Gründer et al., 2024). Shortcomings of this approach are underlined by recent clinical trials that highlight the time-limited character of psychedelic therapy for depression (Carhart-Harris et al., 2021; Goodwin et al., 2022; von Rotz et al., 2023). Only one study, using a waiting list control group (i.e., receiving no treatment at all) suggested improvement beyond 3 months of psychedelic therapy for major depression (Davis et al., 2021). The most recent data from the psilocybin depression trial by COMPASS indicate that after 6 weeks only a 3.6 point difference between the psilocybin and the placebo-group was observed on the MADRS depression scale, which ranges from 0 to 70 – a difference that was considered “clinically meaningful” by COMPASS, while according to the scientific literature a difference of around 3 points cannot be distinguished by clinical raters (Hengartner et al., 2020). It should be noted however, that the COMPASS trials focused on patients who were classified as “treatment-resistant.” A study focusing on non-treatment-resistant MDD patients reported a 12.3-point difference between the psilocybin and the placebo-group on the MADRS at day 43 (Raison et al., 2023), indicating that the prospects for short-term improvement for this less-difficult-to-treat group may be more favorable (for further discussion of this, see: the “Implications: Patient selection, mystical experiences, positive afterglow and psychotherapy” section).

### Comparison with historical reports, meta-analyses and contemporary clinical studies on AUD

An early review of studies about LSD psychedelic therapy for individuals with AUD (Abuzzahab and Anderson, 1971) concluded that the study designs and data gathered were so diverse that they do not allow conclusions about treatment efficacy. A more recent historical review (Bonson, 2018, p. 601) noted that the Kurland et al., clinical studies with LSD in individuals with AUD “are among the best of that era in that they attempted to conform to the regulations for adequate and well-controlled studies.” – but also that “much of the hallucinogen research in this period falls short of ideal scientific methodology” (Bonson, 2018). Key examples of the methodological shortcomings that are listed include the unethical procedures regarding informed consent – which was often limited and mostly to clear the researchers from any liability regarding AEs – and the lack of proper control conditions and blinding. Bonson (2018) cites researchers at NIMH and Spring Grove who at the time already criticized the scientific methodology of the psychedelic trials and she concludes that these early studies can only be used to: “*produce hypotheses that can be subjected to modern scientific evaluation through appropriate regulatory oversight.*”

Interestingly, an often-cited meta-analysis by Krebs and Johansen (2012) came to a quite different conclusion based on the same evidence. Their meta-analysis included six controlled trials (Bowen et al., 1970; Hollister et al., 1969; Ludwig et al., 1969; Pahnke et al., 1970; Smart et al., 1966; Tomsovic, 1970) and concluded that psychedelic therapy appeared an effective and promising intervention for AUD. However, this meta-analysis suffered from methodological and statistical flaws. First, the categorical data on alcohol misuse was dichotomized into “improved” and “not improved” based on unclear criteria. Second, without providing additional justification, the authors “pooled” data for all participants in the LSD treatment groups and all those in the control groups for their analysis (instead of treating the different interventions as different independent groups). They claim that between-trial heterogeneity was assessed by specific statistics and found to be negligible; however, methods and results for this are not given. Thirdly, the Cochrane risk-of-bias tool was used for features as sequence generation, allocation concealment, blinding, incomplete outcome data, selective outcome reporting, but other potential sources of bias were not reported (e.g., suggestive preparation or “no information” about treatment given). Fourthly, the authors used questionable statistical methods which could not be independently reproduced (Sicignano et al., 2024). For these reasons, this meta-analysis and its positive conclusions about the efficacy of psychedelic therapy for AUD have been disputed (for additional methodological concerns, see: Sicignano et al., 2024).

A recent meta-analysis by Sicignano et al. (2024) offers a more comprehensive, systematic and transparent assessment of studies regarding the efficacy of psychedelic therapy for AUD. The authors reviewed six controlled studies, several of which were also included in our review (see Table 2). Taking into account only the three most methodologically stringent and high quality randomized controlled trials (RCTs), they found a significant reduction in drinking or abstinence for the psychedelic compared to the control condition for the first follow-up, but not at 2–3 months or 6-months of follow-up – in line with the results of our narrative review. Also, this meta-analysis reports high heterogeneity in the analyses, likely related to different treatment methods and outcomes between studies. The risk of bias of the included studies was assessed systematically, revealing that some trials had serious limitations, such as small sample sizes or inconsistent reporting of follow-up outcomes. Despite these limitations, the authors conclude that while in the short-term the effects appear favorable for PT, currently there is no evidence for the long-term efficacy of PT.

A recent narrative systematic review also included some of the studies that we reviewed here but refrained from quantifying any of the outcomes, due to study heterogeneity relating to outcome measures and timing of follow-ups (Piper et al., 2025). However, the authors concluded that a recent study by Bogenschutz et al. (2022), a two-center placebo-controlled phase-2 superiority RCT of psilocybin for AUD yielded the best evidence for efficacy of PT. This RCT administered two sessions with either high-dose psilocybin or diphenhydramine (active placebo). Ninety-three patients completed at least one session. The study reported significant differences in favor of psychedelic therapy for reduced heavy drinking days, reduced drinking days and reduced number of drinks per day (measured by self-ratings) for some weeks after the intervention. However, we note that this difference was no longer significant after 5 months and was only based on self-ratings. The study originally intended to collect a valid biomarker for drinking behavior (hair or fingernail ethyl glucuronide; EtG) in week 24, but EtG data were only available for about 53% of the patients and were not reported, which is unfortunate given that it would provide more objective assessments of actual alcohol consumption compared to self-report (for discussion, see: Nielsen et al., 2021).

### Implications: Patient selection, mystical experiences, positive afterglow and psychotherapy

The lack of durable treatment effects in psychedelic therapy could be used as an argument to question the privileged status of the RCT for establishing efficacy and safety (Schenberg, 2021, 2024). As Schenberg argues, the epistemic authority granted to double-blind RCTs may be philosophically biased and ill-suited for psycho-therapeutic interventions in which expectancy, agency and subjective change are not “noise” that needs to be controlled for, but central treatment factors. This view resonates with 4E approaches that also critique simplistic (neuro)reductionist explanations of the efficacy of psychedelic therapy and the underlying causal assumptions by emphasizing that therapeutic change emerges from embodied, embedded, enactive and extended interactions between substances, agents and setting (see, for instance: Kiverstein et al., 2025; Meling and Scheidegger, 2023). A broader sociotechnical view – such as Hauskeller’s work on psychiatry, pharmacological enhancement and the governance of evidence—shows how RCT-based hierarchies of evidence are embedded within institutional, regulatory and commercial arrangements that systematically privilege measurable, short-term symptom change over processual, relational and meaning-laden forms of improvement (Hauskeller, 2025).

While these systematic changes remain important points for future discussion, building on the studies that we discussed in this paper, we argue that an important reason for the lack of long-term effects, both in the early controlled studies and in contemporary clinical trials, may be found in the selection of the patients. The early studies primarily focused on “chronic” AUD – a disorder often characterized by a high amount of psychiatric comorbidity. These chronic patients typically had a history of failing to respond to other therapeutic interventions. In the 1960s, the choice to focus on chronic patients was typical for most studies aimed at evaluating a novel psychoactive substance in therapy. Research on lower-dose *psycholytic* therapy also focused on chronic psychoneurotic patients (Grof, 1980; Passie, 2024). In recent psychedelic clinical trials, we witness a similar tendency to focus on what has been called treatment-resistant disorder. However, the concept of “treatment-resistant” is ill-defined (Conway et al., 2017): in MDD the most common criterion includes not having responded well previously to two antidepressant drugs with prior adequate dose and duration. However, there appears to be no consensus on how this should be assessed and evaluated in scientific and clinical practice (Gaynes et al., 2020). Another problem with this approach is related to the questioned efficacy of conventional antidepressant drugs (Kirsch, 2009; Moncrieff, 2008).

In contrast to the current focus on this ill-defined, operationalized and potentially difficult to treat group of treatment-resistant patients, more lasting positive results with respect to reducing depression and anxiety have been observed with psychedelic therapy in patients with a life-threatening diagnosis (Gasser et al., 2014; Griffiths et al., 2016; Grob et al., 2011; Grof et al., 1973; Ross et al., 2016). This group of patients usually does not have a chronic mental illness, but suffers from reactive anxiety, depression and social isolation induced by the acute/sub-acute impact of a life-threatening diagnosis or impeding death. Their better psychological health on average suggests a stronger resilience, and better social and salutogenetic resources – although currently some of the reported positive benefits can also be attributed to the more frequent intensive therapy sessions provided in the psychedelic condition. The hypothesis that this specific group of patients are better suited to benefit from psychedelic therapy compared to the ill-defined group of “treatment-resistant” patients deserves systematic evaluation in future controlled studies.

A recurring topic throughout this review is the question of to what extent mystical peak experiences are therapeutically significant. Many studies, reviews and meta-analyses have consistently pointed out that the intensity of the mystical experience – typically measured through the mystical-experience questionnaire – correlates positively with better therapeutic outcomes (Griffiths et al., 2006, 2011; Kangaslampi, 2023; Ko et al., 2022). The explanation for this observed association is often found in the profound feelings of meaning and significance that individuals report after a psychedelic experience (i.e., “the most meaningful experience of my life”), and the resulting insights that can prove beneficial if integrated in one’s daily life. It has also been found that psychedelic-induced mystical experiences in non-clinical populations can have a positive influence on the personality dimension “openness” (MacLean et al., 2011), which is also considered favorable for psychotherapeutic processing. We do not contest that psychedelic-induced mystical experiences *can* be therapeutically meaningful, but that if they are not embedded within a strong psychotherapeutic framework, the insights and therapeutic benefits will be rather ephemeral at best (for further discussion, see: Van Elk, 2025). There are also considerable risks that come with the currently single-sided focus on mystical experiences in the psychedelic literature including (a) overlooking other subjective effects beyond mystical experiences that can be therapeutically meaningful (Yaden et al., 2024), (b) the risk of false insights, as in a mystical experience everything is tagged with “significance” (Jylkkä, 2025) and (c) disappointment as patients may not get the “peak-experience” they were hoping for (van Elk and Fried, 2023).

Instead, the rather short-lived nature of the therapeutic effects of psychedelic therapy in the mostly chronically mentally ill patients could also be phrased more positively in terms of a “positive mood state for some time after the drug session.” This after-effect has been described as a “psychedelic afterglow” (Evens et al., 2023). During such a state, some patients experience better mood and apparently are in a more open and flexible psychological state (MacLean et al., 2011; Sherwood et al., 1962). The results from recent RCTs focusing on psychedelic therapy for MDD are also in line with the notion of a positive afterglow for some weeks or months (Carhart-Harris et al., 2021; Goodwin et al., 2022; Palhano-Fontes et al., 2019). This positive afterglow underscores the importance of additional psychotherapy sessions to induce lasting changes in psychological functioning (see also: Gründer et al., 2024). Some psychedelic therapy researchers of the early controlled trials reviewed here, already suggested that to enhance the durability of improvements the therapist needs to use the post-psychedelic period, when the patient is in a more open and flexible psychological state for psychotherapeutic processing (Grof, 1980; Savage et al., 1964; Sherwood et al., 1962). For instance, Pahnke et al. (1970; p. 862), director of psychedelic therapy research at the *MPRC* and major proponent of the psychedelic therapy paradigm, concluded:

LSD is not a substitute for skilled psychotherapy. Experiments where LSD was used primarily as a chemotherapy, or with a minimum of psychotherapy, have not shown any greater efficacy of therapeutic outcome . . . than control groups.

As early European psycholytic work has shown in the 1960s (Mascher, 1967), genuine long-term psychotherapeutic frameworks for the use of LSD-like psychedelics are available and their application might produce longer-lasting and durable betterments (see also: Aicher et al., 2024, 2025; for a comparison between psychedelic (peak) therapy and psycholytic therapy, see: Table 4). Lower doses used in psycholytic therapy produce a different array of experiences, for instance regression, catharsis and psychodynamic activations and insights, which are worked through in conventional psychotherapy sessions (cf., Passie et al., 2022). The drug-centered approach that appears at the core of the psychedelic therapy paradigm (in the past as well as today), focusing on acute and sub-acute neurobiological/psychological drug effects, may rest on an incorrect assumption about the way how to use psychedelics therapeutically and their mechanisms of action. Instead, we suggest that these substances are essentially “psychotherapy-promoting drugs.” They can facilitate psychotherapy but cannot substitute it.

## Concluding remarks

Our review of the early controlled trials of psychedelic therapy indicates that the researchers at that time already tried to address methodological problems by using improved and refined study designs. These studies consistently showed that inducing a peak experience by giving a high dose of psychedelics does not lead to durable improvements for individuals suffering from chronic conditions such as AUD, SUD or psychoneuroses (with symptoms of depression or anxiety). These findings are corroborated by results of recent clinical trials (see e.g., Waldron, 2025) and meta-analyses, indicating that one or two high-dose sessions with a focus on a mystical experience will not produce long-term improvements. Apparently, classical psychedelics such as LSD can support psychotherapy, but they cannot replace it.

## Notes

1. Since the mid-1960s *psychedelic therapy* was also named “*Psychedelic Peak Therapy*” by some of its major proponents ( ) as “. . . the aim is to achieve a positively profound and insightful experience, hopefully yielding beneficial personality and attitudinal changes” (Kurland et al., 1971, 92/3). For consistency, throughout this manuscript we refer to this treatment by its full name, psychedelic therapy and reserve the term psycholytic therapy for the low-dose, multi-session approach (see Table 4).

2. Recently, selective disclosure of drug information in the informed consent has been suggested as a viable alternative for mitigating the breaking blind problem (Aday et al., 2022), but it raises additional ethical concerns (Matvey et al., 2025).
